# Web-based exercise versus supervised exercise for decreasing visceral adipose tissue in older adults with central obesity: a randomized controlled trial

**DOI:** 10.1186/s12877-020-01577-w

**Published:** 2020-05-12

**Authors:** Marcel Ballin, Andreas Hult, Sabine Björk, Emmy Lundberg, Peter Nordström, Anna Nordström

**Affiliations:** 1 Department of Community Medicine and Rehabilitation, Unit of Geriatric Medicine, Umeå University, 901 87 Umeå, Sweden; 2 Department of Public Health and Clinical Medicine, Section of Sustainable Health, Umeå University, Umeå, Sweden; 3 Department of Community Medicine and Rehabilitation, Section of Sports Medicine, Umeå University, Umeå, Sweden; 4 Department of Nursing, Umeå University, Umeå, Sweden; 5 School of Sport Sciences, UiT The Arctic University of Norway, Tromsø, Norway

**Keywords:** Visceral fat, Obesity, Physical activity, Interval training, Ageing, eHealth

## Abstract

**Background:**

Visceral adipose tissue (VAT) is a strong risk factor for cardiovascular disease and increases with age. While supervised exercise (SE) may be an effective approach, web-based exercise (WE) have other advantages such as being more readily accessible. Therefore, we evaluated the effects of WE on VAT, body composition and cardiometabolic risk markers in centrally obese older adults and compared the effects of WE to SE. We also explored the feasibility of WE.

**Methods:**

In a randomized controlled trial conducted in Umeå, Sweden during January 2018 – November 2018, *N* = 77, 70-year-old men and women with central obesity (> 1 kg VAT for women, > 2 kg for men) were randomized to an intervention group (*n* = 38) and a wait-list control group (*n* = 39). The intervention group received 10 weeks of SE while the wait-list control group lived as usual. Following a 10-week wash-out-period, the wait-list control group received 10 weeks of WE. The primary outcome was changes in VAT. Secondary outcomes included changes in fat mass (FM), lean body mass (LBM), blood lipids, fasting blood glucose. Additionally, we explored the feasibility of WE defined as adherence and participant experiences.

**Results:**

WE had no significant effect on VAT (*P* = 0.5), although it decreased FM by 450 g (95% confidence interval [CI], 37 to 836, *P* < 0.05). The adherence to WE was 85% and 87–97% of the participants rated aspects of the WE intervention > 4 on a scale of 1–5. Comparing SE to WE, there was no significant difference in decrease of VAT (Cohen’s *δ* effect size [ES], 0.5, 95% CI, − 24 to 223, *P* = 0.11), although SE decreased FM by 619 g (ES, 0.5, 95% CI, 22 to 1215, *P* < 0.05) compared to WE.

**Conclusions:**

Ten weeks of vigorous WE is insufficient to decrease VAT in centrally obese older adults, but sufficient to decrease FM while preserving LBM. The high adherence and positive experiences of the WE intervention implies that it could serve as an alternative exercise strategy for older adults with central obesity, with increased availability for a larger population.

**Trial registration:**

ClinicalTrials.gov (NCT03450655), retrospectively registered February 28, 2018.

## Background

The pandemic of physical inactivity and obesity is not limited to young and adult populations but is highly prevalent also in older populations [1–4]. Together, these are strong and well-established risk factors for adverse outcomes including cardiovascular disease (CVD), type 2 diabetes and mortality [5–8]. Specifically, central obesity which is characterized by an excessive accumulation of visceral adipose tissue (VAT) has been shown to be a stronger risk factor than general obesity [7, 9, 10]. Considering that VAT increases with age [11], it may be particularly important to aim to decrease VAT in older people experiencing central obesity.

Older people stand to gain several health benefits from regularly performing different types of exercise [12]. However, as a result of the economic burden due to physical inactivity, obesity and CVD [13–15] combined with the growth of the older population [16], innovative solutions for tackling these problems on a population level are needed. In light of the increasingly digitalized healthcare [17], digital and web-based health interventions may be the way forward. These are more cost-efficient and readily accessible [18–20] and therefore has the potential for wide-spread distribution of exercise interventions in home-based settings. Previous systematic reviews have described the positive effects of web-based physical activity interventions [21, 22]. In conjunction with the increasing number of older internet-users during the past two decades [23, 24], positive effects have been seen also in older people specifically [25, 26]. However, the feasibility and effectiveness of vigorous web-based exercise (WE) for older adults with central obesity have to the best of our knowledge not been established.

We have previously shown that 10 weeks of vigorous supervised exercise (SE) improved body composition and cardiometabolic risk markers in older adults with central obesity [27, 28]. Against this background, the primary aim of the present study was to investigate whether 10 weeks of vigorous WE is sufficient to decrease VAT in centrally obese older adults, and compare the effects of WE to the effects from the previous SE intervention. Additional outcomes were measures of body composition and cardiometabolic risk markers. Finally, we also aimed to explore the feasibility of WE in terms of adherence and participant experiences.

## Methods

### Study design

**Fig. 1:**
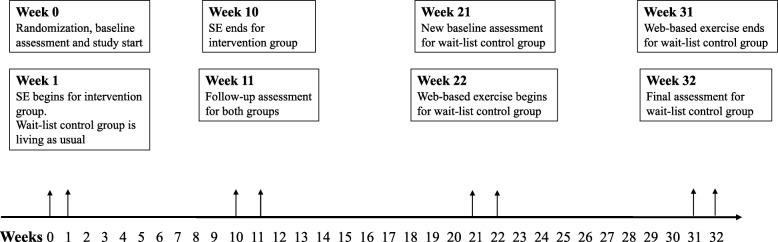
Overview and timeline of the study, baseline- and follow-up assessments, and delivery of interventions

This study was a two-armed randomized controlled trial conducted in Umeå, Sweden, during January 2018 – November 2018. Participants were randomized 1:1 to an intervention group or a wait-list control group (ClinicalTrials.gov registration no. NCT03450655). An overview and timeline of the study, follow-up assessments and delivery of interventions is presented in Fig. 1 Following randomization and baseline assessment, the intervention group received SE for 10 weeks at a university hospital research clinic, while the wait-list control group lived as usual. After this initial 10-week phase, both groups underwent follow-up assessment at the research clinic. The 10-week results of the trial have been published previously [27, 28]. Next, the wait-list control group underwent a 10-week wash-out period, after which they returned to the research clinic for another reassessment, which served as baseline in the wait-list control group’s intervention. The wait-list control group’s intervention was 10 weeks of WE, after which they returned for a final follow-up assessment. To compare the effects of the two interventions, changes in the outcome variables during weeks 0–10 in the SE group were compared to changes in the outcome variables during weeks 20–30 in the WE group

The methods, procedures, design and informed consent protocol for the present trial were reviewed and authorized by the regional research ethical review board of Umeå (Ref. 2017/521–31). The trial was conducted correspondingly to the World Medical Association’s Declaration of Helsinki and reporting of the trial was performed according to the CONSORT guidelines [29].

### Participants

Participants were community-dwelling 70-year-old men and women recruited during January 2018 – February 2018 from the ongoing primary prevention study Health Ageing Initiative (HAI), described in detail previously [30]. In short, all 70-year-olds living in Umeå municipality are contacted using population registers and offered to participate in an extensive health survey. As part of the HAI protocol, several measurements are made including body composition, bone density, physical activity, blood pressure and blood markers among others. In addition, all participants are given tailored health recommendations and motivational interviewing with focus on physical activity and diet. So far, around 70% of all eligible participants have chosen to participate [30].

### Inclusion and exclusion criteria

To be included in the present study, participants were required to be centrally obese defined as at least 1 kg and 2 kg of VAT mass for women and men respectively and also pass a resting electrocardiogram (ECG; Schiller Cardiovit CS-6/12; Schiller AG). These cutoff-values for VAT were based on previous dual-energy X-ray absorptiometry (DXA) measures from > 2600 participants in the HAI-study that were centrally obese defined by waist circumference [31] (> 88 cm for women and > 102 cm for men).

All individuals with conditions that contraindicated training or affected the ability to perform the training program were excluded. Specifically, these exclusion criteria were as follows: physical disabilities; myocardial infarction or stroke during the previous 12 months; blood pressure higher than 175/100 mmHg; heart conditions that may worsen through intense exercise e.g. angina pectoris.

### Sample size

Statistical power was calculated based on data on VAT mass from 1200 HAI-participants (606 women and 594 men) with central obesity, defined as above. The calculations showed that a total of 33 men or 45 women per group, with somewhere in between that range for mixed groups, would provide 80% power to detect a 20% reduction in VAT, with the alpha level set to 0.05. The analyses were performed separately for men and women due to the uneven VAT mass distribution between sexes. The calculations were performed using G*Power version 3.0.10 [32].

### Randomization

Following baseline assessment, all participants were randomized using 80 pre-prepared and shuffled opaque sealed envelopes. Enclosed were notes indicating “Exercise” or “Control” (40 each) where the former meant allocation to the SE group and the latter meant allocation to the WE group. MB and EL were in charge of the randomization and all envelopes were shuffled before each participant was given permission to draw an envelope to ensure allocation concealment.

### Intervention

Participants randomized to WE received a web-based weekly progressive interval training program which was recorded by MB and EL using a Nikon D5600 digital camera and edited using Corel VideoStudio X8 (Corel Corp. Ottawa, CA) before uploaded to a website (www.healthyageinginitiative.com/kondition). The training program was delivered in terms of 10 weekly videos of training sessions where each video represented one week of the intervention and would be viewed three times weekly. For the recording of these videos, a female and male participant from the SE group were the actors showcasing the program. Please see Additional file 1 for an example of the training videos. There was also a general informative video and instructional videos for all exercises included in the training program which were showcased by MB and EL. The general informative video included information regarding exercise and central obesity in relation to cardiovascular health; detailed description of the exercise program; and safety information. Specifically, participants were prompted to contact the National e-Health Service (1177 Healthcare Guide’s e-service Support and Treatment) if they perceived any discomfort. The exercises showcased in the instructional videos were dynamic body-weight exercises designed to allow individual progression and adjustments (Additional file 2). Specifically, participants could choose to perform an easier variation or a more challenging variation of each exercise. In addition, they were instructed to adjust the tempo and range of motion in the exercises to match their individual capacity. A detailed description of all exercise is available in Additional file 3. Work-to-rest ratio during the intervals was 40 s/ 20 s and the intervals were to be performed at a vigorous level of intensity, prescribed using a modified scale for rate of perceived exertion (RPE), based on the Borg CR10 scale [33]. Volume increments were applied simultaneously for all participants in terms of progressively adding the number of working sets throughout the 10 weeks of training, thus increasing the training duration. Initially, the duration of interval training was 18 min, with a gradual increment to a final maximum duration of 36 min. In the week before the start of the intervention, all participants in the WE group were invited to partake in one supervised session led by MB and EL with the aim of providing the participants with practical advice regarding performance of the exercise program and clarifying potential ambiguities. All participants were then given a suspension band that was required for a few of the exercises, and they were encouraged to use the bands for support of balance when necessary to reduce the risk of falling. Finally, AH presented the website and intervention material to all participants and provided point-by-point instructions in how to access and use the website and intervention material.


**Additional file 1.** Example of the weekly videos with web-based exercise sessions.



**Additional file 2.** Example of the instructional videos of the exercises used in the training program and how these could be adjusted.


Participants randomized to the SE group performed the exact same training program at a university hospital research clinic in groups of 8–10 participants under supervision from two fitness instructors (MB and EL). Volume increments were identical to those in the WE group.

### Assessment procedures

All assessments were performed during daytime, with the ambition of standardizing and matching the exact time-of-day of assessments as far as possible. The research nurses in charge of assessments were blinded at all occasions apart from the final follow-up where blinding was not possible. All participants were informed to refrain from exerting any intense physical activity or consuming alcohol the day prior to each assessment, while being in a fasted state for at least four hours. Participants self-reported medication, smoking, diabetes, and any history of myocardial infarction or stroke. Blood pressure was measured using the digital automatic blood pressure device Omron M6 Comfort HEM-7221-E (Omron Healthcare, Kyoto, Japan) after a 15-min rest with the subjects in a supine position. Two measurements were made whereof the lowest one was recorded. Data on daily physical activity were obtained from the HAI-study where the participants physical activity were objectively assessed during one week of registration using Actigraph GT3X+ accelerometers (Actigraph, Pensacola, FL, USA), described in depth elsewhere [30].

### Primary outcome

The primary outcome for this trial was the mean change in VAT mass (grams), measured using a Lunar iDXA device and the CoreScan application (GE Healthcare Lunar, Madison, WI, USA). The CoreScan application utilizes algorithms which distinguishes VAT from subcutaneous fat mass, thereby allowing quantification of VAT. Trials using iDXA CoreScan measurements have previously reported a test-retest precision error (RMSSD; root mean square standard deviation) of 41.4 g VAT in overweight people [34].

### Secondary outcomes

#### Body composition

From the same scan using the Lunar iDXA device, measures of total fat mass (FM, grams), body fat percentage (BFP, %) and total lean body mass (LBM, grams) were derived. The percent coefficient of variation using iDXA has been deemed 1.8% for FM and 0.8% for LBM in overweight people [34]. Following assessment of body weight and height using a digital scale (HL 120; Avery Berkel, Fairmont, MN, USA) and a gauge (Holtain Limited; Crymych, Dyfed, UK), the body mass index (BMI, kg/m^2^) was calculated by dividing body weight by height squared.

#### Cardiometabolic blood markers

Total cholesterol (TC, mmol/l), high-density lipoprotein cholesterol (HDL, mmol/l), low-density lipoprotein cholesterol (LD, mmol/l), triglycerides (TG, mmol/l) were collected by venipuncture by the research nurses and subsequently sent for analysis at the accredited laboratory at the Department of Clinical Chemistry, Umeå University Hospital. Fasting blood glucose (FBG, mmol/l) was measured using the HemoCue 201 RT system (Radiometer Medical ApS, Denmark).

#### Feasibility

Adherence to the WE was evaluated by having the participants self-report the number of completed training sessions throughout the intervention using a protocol provided to them prior to the intervention. To explore the participants’ experiences, all participants in the WE group filled in a survey during the final follow-up assessment including four questions based on a 5-point Likert Scale (1 = not at all satisfied/not likely at all and 5 = very satisfied/very likely). The questions were as follows: 1) “How satisfied are you with the information provided to you before the start of the intervention?” 2) “How satisfied are you with the design of the exercise-videos?” 3) “How satisfied are you with the sample of exercises?” 4) “How likely is it that you will continue with this type of training?

### Statistical analysis

Data was inspected visually through histograms to assess normal distribution. Tests for significant differences between the groups prior to their interventions were performed using χ^2^-tests on categorical variables and *t*-tests for independent samples on continuous variables as data was deemed normally distributed. Within-group changes in the outcome variables were analyzed using *t*-tests for paired samples following that data was assumed normal in distribution. The adherence to WE was presented as median and interquartile range (IQR) and participant experiences based on the survey answers were presented as percentage.

The differences in changes in outcome variables from pre- to post intervention between the WE- and SE group were assessed using a series of analyses of covariance (ANCOVAs) with adjustments for baseline values. Effect sizes (ES) for between-group differences were calculated using Cohen’s *δ* [35]. All analyses were conducted on an intention-to-treat basis using SPSS software v25.0 (IBM Corp. Armonk, NY), with the significance level set at *P* ≤ 0.05 for all analyses.

## Results

### Participant flow

**Fig. 2:**
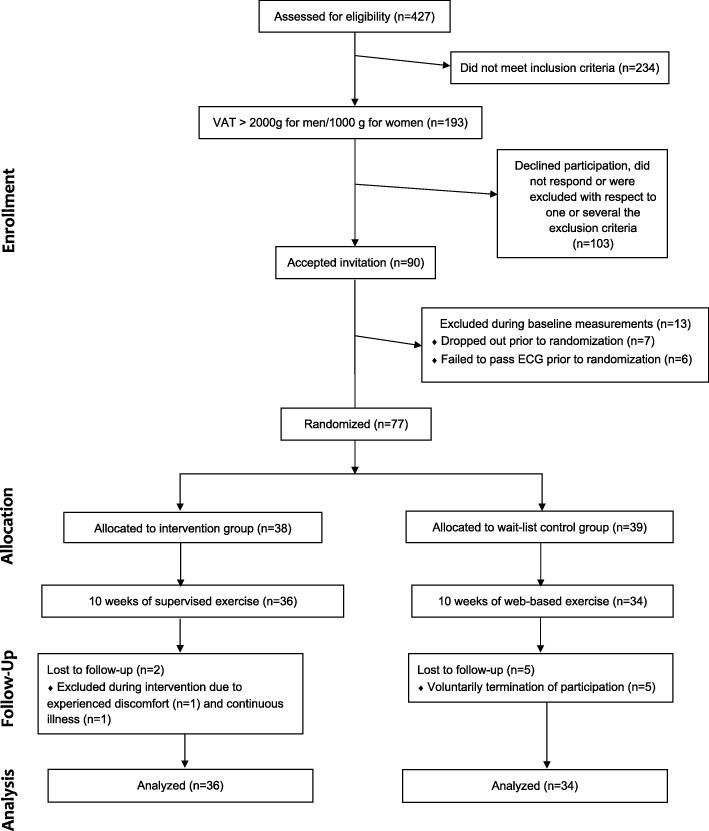
Study flow chart

From the 90 eligible individuals, seven individuals were absent during baseline assessments and another six individuals did not pass the resting ECG, resulting in exclusion. Eventually, *N* = 77 individuals were randomized to SE (*n* = 38) and WE (*n* = 39). Two participants in the SE group were unable to complete the trial and were excluded because of discomfort following training sessions (*n* = 1) and illness unrelated to the present intervention (n = 1). A total of five participants in the WE group withdrew from the study. Of these, three withdrew between week 0 and week 11, while the other two withdrew between week 21 and week 32. Consequently, 36 and 34 participants in the SE and WE group respectively completed the trial. A detailed figure of the participant flow is available in Fig. 2.

### Participant characteristics prior to intervention

Participant characteristics are presented in Table 1. Both groups were similar on all variables except age and blood pressure. Mean VAT mass prior to the intervention was 2.10 ± 0.95 kg and 2.30 ± 0.82 kg for the WE group and the SE group respectively. Mean BMI prior to the intervention was 28.7 ± 3.5 kg/m^2^ and 28.7 ± 3.1 kg/m^2^. The sex distribution was similar in both groups (53% women in the WE group).

**Table 1:** Participant characteristics prior to interventions

**Variables**	**SE*n*** **= 38**	**WE*n*** **= 36**	***P***
Age (years)	70.7 ± 0.25	71.3 ± 0.24	< 0.001
Women	18 (47)	19 (53)	0.6
Current smoker^a^	2 (6)	0	0.2
Height (cm)	169 ± 9	171 ± 10	0.3
Weight (kg)	84.7 ± 9.4	84.1 ± 12.6	0.8
Waist circumference (cm)	103 ± 8.4	103 ± 8.5	0.8
Systolic blood pressure (mmHg)	143 ± 15	128 ± 15	< 0.001
Diastolic blood pressure (mmHg)	84 ± 7	80 ± 6	0.005
**Body composition**			
VAT (kg)	2.30 ± 0.82	2.10 ± 0.95	0.3
FM (kg)	33.36 ± 5.82	32.78 ± 5.86	0.7
BFP (%)	39.6 ± 6.6	39.2 ± 5.7	1.0
LBM (kg)	48.56 ± 8.73	48.66 ± 9.52	1.0
BMI (kg/m^2^)	29.7 ± 3.1	28.7 ± 3.5	0.19
**Daily PA**^**a**^			
Sedentary (%)	66.0 ± 7.9	65.9 ± 10.2	1.0
Light PA (min)	252.2 ± 68.9	250.3 ± 73.5	0.9
Moderate PA (min)	27.2 ± 20.1	29.2 ± 26.1	0.7
Vigorous PA (min)	0.2 ± 0.8	0.2 ± 0.7	0.7
Total steps	6341 ± 2450	6770 ± 3318	0.6
AEE (kcal)	373 ± 159	366 ± 150	0.9
**Medical history and medication**			
Lipid-lowering medication	13 (34)	18 (50)	0.2
Antihypertensive medication	23 (61)	20 (56)	0.8
Type 2 diabetes^a^	4 (11)	6 (15)	0.5
Previous stroke^a^	1 (3)	1 (3)	0.9
Previous myocardial infarction^a^	2 (5)	4 (10)	0.5

Data are presented as group means ± standard deviation or number (percentage)

Abbreviations: *AEE* activity energy expenditure; *BFP* body fat percentage; *BMI* body mass index; *FM* fat mass; *LBM* lean body mass; *PA* physical activity; *SE* supervised group-exercise; *VAT* visceral adipose tissue; *WE* web-based exercise

^a^Baseline data from study start following randomization

### Effects and feasibility of the web-based exercise intervention

**Fig. 3:**
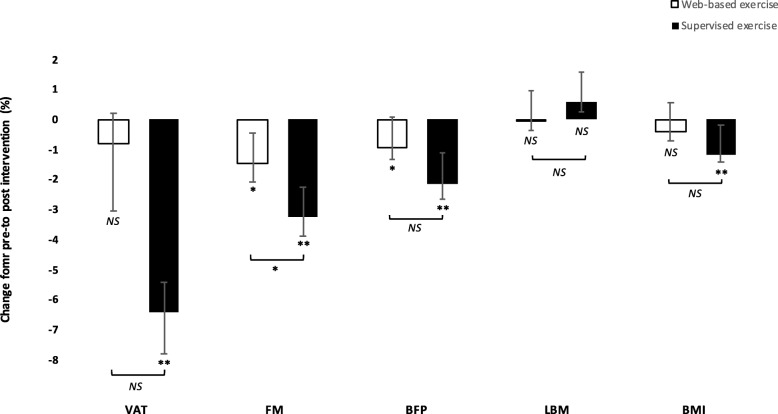
Mean percental changes in body composition following 10 weeks of web-based exercise (open bars) vs 10 weeks of supervised exercise (filled bars). Error bars represent standard errors of the mean. BFP indicates body fat percentage; BMI, body mass index; DBP, diastolic blood pressure; FM, fat mass; LBM, lean body mass; NS, not significant; SBP, systolic blood pressure; VAT, visceral adipose tissue * indicates *P* < 0.05, ** indicates *P* < 0.001

The WE intervention had no significant effect on the primary outcome VAT from pre- to post-intervention, although FM decreased by 450 g (95% confidence interval [CI], 37 to 836, *P* < 0.05), corresponding to a 1.4% decrease in FM, and BFP decreased from 39.0 ± 5.8% to 38.7 ± 6.0% (95% CI, 0.1 to 0.6, *P* < 0.05) (Table 2, Fig. 3). WE had no significant effect on the remaining outcomes (Table 2). The median adherence was 85% (IQR, 59–100%). Regarding experiences of the intervention, the questions asked (scale 1–5) at the follow-up assessment showed the following result: a) 97% rated the information prior to the intervention > 4 b) 94% rated the design of the exercise-videos > 4 c) 87% rated the sample of exercises > 4 d) 81% answered > 4 as for the probability that they would continue with this type of training.

**Table 2:** Changes in outcomes following 10 weeks of supervised exercise and 10 weeks of web-based exercise

	**Changes within groups**						**Comparison in changes between groups**		
	**SE*n*** **= 36**			**WE*n*** **= 34**			**SE*n*** **= 36**	**WE*n*** **= 34**	
	**Baseline**	**10 Weeks**	***P***	**Baseline**	**10 Weeks**	***P***	**Mean change (95% CI)**	**Mean change (95% CI)**	***P*for difference**
Body composition^a^									
VAT (g)	2339 ± 809	2176 ± 758	< 0.001	2061 ± 844	2025 ± 829	0.5	− 163 (− 232 to − 94)	− 36 (− 140 to 67)	0.11
FM (g)	33,424 ± 5940	32,353 ± 6004	< 0.001	32,313 ± 5573	31,863 ± 5752	0.034	− 1071 (− 1504 to − 638)	−450 (− 863 to − 37)	0.042
BFP (%)	39.6 ± 6.6	38.8 ± 6.9	< 0.001	39.0 ± 5.8	38.7 ± 6.0	0.022	− 0.8 (− 1.2 to − 0.4)	−0.3 (− 0.6 to − 0.1)	0.051
LBM (g)	48,621 ± 8837	48,900 ± 8918	0.08	48,385 ± 9359	48,325 ± 9090	0.7	280 (−35 to 594)	−60 (− 387 to 266)	0.13
BMI (kg/m^2^)	29.8 ± 3.1	29.4 ± 3.1	< 0.001	28.5 ± 3.5	28.4 ± 3.5	0.2	− 0.4 (− 0.5 to − 0.2)	−0.1 (− 0.3 to 0.1)	0.072
Blood markers (mmol/l)^b^									
TC	5.18 ± 1.25	4.93 ± 1.17	0.006	5.20 ± 0.92	5.11 ± 0.89	0.4	−0.25 (− 0.42 to − 0.08)	−0.09 (− 0.32 to 0.13)	0.2
HDL	1.40 ± 0.42	1.39 ± 0.36	0.7	1.54 ± 0.43	1.54 ± 0.39	0.8	−0.01 (− 0.07 to 0.05)	−0.01 (− 0.06 to 0.05)	0.4
LDL	3.08 ± 1.09	2.89 ± 0.99	0.010	2.95 ± 0.90	2.88 ± 0.89	0.5	−0.18 (− 0.31 to − 0.05)	−0.07 (− 0.25 to 0.12)	0.4
TG	1.55 ± 0.45	1.47 ± 0.42	0.3	1.58 ± 0.57	1.53 ± 0.56	0.5	−0.08 (− 0.22 to 0.06)	−0.05 (− 0.21 to 0.11)	0.7
FBG	5.98 ± 1.09	5.79 ± 0.67	0.13	5.94 ± 1.14	5.99 ± 1.09	0.5	−0.19 (− 0.44 to 0.06)	0.06 (− 0.12 to 0.23)	0.067

^a^ Data on body composition for the SE group was originally published in ‘Journal of the American Geriatrics Society 2019,67 [8]:1625–1631’ by John Wiley & Sons Ltd., from whom permission was granted to reuse the data in the present study

^b^ Data on blood lipids for the SE group was originally published in ‘Clinical Interventions in Aging 2019,14:1589–1599’ by Dove Medical Press Ltd., from whom permission was granted to reuse the data in the present study

All data are presented as means ± standard deviation at baseline and follow-up for within-group changes with *P*-values derived from paired *t*-tests. For comparison between groups, mean change and 95% CI from within each group is presented with *P*-values for between-group difference derived from ANCOVA adjusted for baseline values

Abbreviations: *BFP* body fat percentage; *BMI* body mass index; *CI* confidence interval; *FBG* fasting blood glucose; *FM* fat mass; *HDL* high-density lipoprotein cholesterol; *LBM* lean body mass; *LDL* low-density lipoprotein cholesterol; *SE* supervised exercise; *TC* total cholesterol; *TG* triglycerides; *VAT* visceral adipose tissue; *WE* web-based exercise

### Web-based exercise compared to supervised exercise

Comparing the effects of the interventions, there was no statistically significant difference in decrease of VAT mass, although the SE group decreased VAT by 100 g (ES, 0.5, 95% CI, − 24 to 223) compared to the WE group. Moreover, the SE group decreased FM by 619 g (ES, 0.5, 95% CI, 22 to 1215, *P* < 0.05), compared to the WE group. No significant differences on the remaining outcomes were observed (Table 2, Fig. 3).

### Adverse events and other effects of the training

Five participants in the SE group reported disorders in the musculoskeletal system of which none lasted longer than the intervention period or affected participants’ ability to complete the outcome assessment. No adverse events in the WE group were reported. Several participants in both groups who had osteoarthritis preceding the start of this trial reported pain relief during the course of the intervention**.** A few participants in the WE group stated that in the beginning of the intervention it was somewhat difficult to reach the prescribed intensity.

## Discussion

The main findings of the present study are that 10 weeks of WE is insufficient to decrease VAT, but sufficient to significantly decrease FM. Moreover, the adherence to WE was high and the participants’ experiences were overwhelmingly positive. While SE is shown to lead to relatively larger effects in the short term, the results of the present study suggest that WE could serve as an alternative exercise strategy for older adults with central obesity, with increased availability for a greater number of individuals.

To our knowledge, this is the first study to evaluate the effects of vigorous WE on VAT in older adults. We argue that the lack of effect may partly be explained by an insufficient intervention duration characterized by lack of high intensity. This has some support from previous research where longer interventions seem preferable for improving body composition [36] and higher intensities could potentially be more favorable for decreasing VAT [37]. While the duration of the intervention was identical in both groups, the larger decrease in VAT in the SE group could partly be explained by the presence of supervisors facilitating for them to quickly learn how to exert a high intensity, as opposed to the WE group which was unsupervised and most likely had an extended learning period. Furthermore, the lack of effect on VAT within the WE group as related to intensity could also be related to discrepancies in motivation between the groups, given that SE was prescribed immediately after randomization in contrast to the WE which was prescribed at a much later stage. As a result, this would imply that effects of WE may have been slightly underestimated. In order to gain a more detailed and valid understanding of the effects of WE on VAT, and how it compares to SE also in the long term, parallel randomized-controlled trials with a longer intervention period and additional long-term follow-up assessments are needed.

However, an encouraging finding was that WE significantly decreases FM and BFP, which has clinical significance given that excess FM is casually associated with cardiovascular outcomes and type 2 diabetes [38, 39]. To which extent a given amount of decreased FM reduces the risk of CVD has however not yet been established, and remains an area for future investigation [40]. When considering the present findings in relation to previous research, a study by Wijsman and colleagues observed a somewhat larger decrease in BFP following their web-based intervention [26]. However, it should be taken into consideration that the WE group in our study had seen some positive effects already during their time as a control group [27]. Given the association between baseline values and the subsequent rate of decrease, as well as decreases being larger in the early phase of interventions [41], the observed decrease in FM and BFP may therefore be slightly underestimated. Likewise, it could be assumed that if the intervention was prescribed immediately following randomization, larger effects may have been observed, although this needs to be confirmed by future studies. Another important finding was that the WE intervention managed to preserve LBM while at the same time decrease FM significantly. While weight loss and dietary interventions are common key-components in obesity-therapy [42] they are often accompanied by loss of LBM which poses serious risks for older adults specifically [43]. Thus, when aiming to decrease obesity in older people it is critical to prescribe adequate exercise to preserve LBM.

Looking at the cardiometabolic blood markers, WE had no effect on lipids and FBG. This is in contrast to a previous study which demonstrated positive effects of WE on FBG and TC [44]. However, the participants were adults with type 2 diabetes, which would explain the beneficial effects of the interventions given that exercise is more effective for improving metabolic outcomes in those with initially higher values [45]. In the study on older adults by Wijsman and colleagues, there were small, albeit significant, effects of their WE intervention on FBG and lipids, possibly due to the greater weight-loss in their study compared to ours [26]. Given the inconsistent results, additional randomized controlled trials including older adults with hyperglycemia and dyslipidemia are required in order to establish the effectiveness of WE on cardiometabolic blood markers in older adults.

In terms of feasibility, we observed a high adherence to WE (85%), which is similar to the adherence to the SE intervention [27]. This resemblance is supported by the findings from a recent systematic review [46] and may have several explanations. For instance, promoting feelings of relatedness is critical when designing training programs for older adults in order to promote adherence [47]. The use of digital training peers may have been beneficial for this cause, which has some support from previous research demonstrating that obese people preferred training peers with similar characteristics [48]. Furthermore, the freedom and flexibility to exercise whenever it suits best, and the possibility to choose an appropriate level of difficulty, are important factors to promote feelings of autonomy [47]. Together, it is further possible that these factors were reflected in the positive ratings of the experiences of the intervention. From here on, we believe that in order to maintain long-term adherence to WE, future interventions should aim to further target factors associated with adherence [49, 50], and incorporate behavior change techniques to promote the effectiveness of the interventions [51]. Specifically, in terms of digital solutions there are indications that mobile applications are beneficial for promoting behavior change also in older adults [52].

The major limitation of the present trial is that the interventions were not initiated at the same time, thus impeding optimal comparisons between the two and possibly underestimating the effects from WE. Moreover, dietary habits were not monitored. There were also limitations related to self-reporting. Specifically, exercise intensity was prescribed using a subjective RPE-scale, making it difficult to determine to which extent participants reached the prescribed intensity. This in turn could then have influenced the observed large inter-individual variability in effect among the participants. Furthermore, the adherence to WE may be overestimated given that it was self-reported, potentially resulting in a lower training volume which in turn would have affected the outcomes as well. Finally, as a result of the number of excluded subjects during baseline assessments, lack of statistical power may have affected the results of the statistical analyses.

The major strengths of the present trial include the randomized designed and the use of DXA for assessment of VAT and body composition. The design of the exercise interventions is another component worth highlighting. Only a handful of easy-to-perform exercises were used, designed to safely fit the current population and a home-environment while still engaging large muscle groups and allowing both progression and individual adjustment without the use of expensive gym-equipment. These are factors previously known to facilitate engagement in exercise among older adults [53, 54]. Also, no adverse events related to the WE intervention was reported which is positive given that fear of injuries is a barrier to exercising among older people [53]. Together, these factors increase the external validity of the findings, and the fact that the WE intervention has the potential to be widely distributed to older people in a home environment could have important public health implications.

## Conclusion

In summary, the main findings of this study are that 10 weeks of vigorous WE is insufficient to decrease VAT in centrally obese older adults, but sufficient to decrease FM while at the same time preserving LBM. There was a high adherence to the WE intervention and the participants’ experiences were overwhelmingly positive. While SE leads to relatively larger effects than WE in the short-term, WE still significantly decreases FM, which has been associated with reduced risk of type 2 diabetes and CVD. Together, these findings indicate that compared to SE, WE could serve as an alternative exercise strategy for older adults with central obesity, with increased availability for a greater number of individuals. This in turn would be of interest to practitioners and researchers within the field of geriatric medicine, public health and health innovation. Given that the present study to our knowledge was the first to investigate the short-term effects of WE on VAT in older adults with central obesity, questions remain regarding whether larger effects could be observed in the long-term.

## Supplementary information

**Additional file 3.** Written description of the exercises used in the training program.

## Data Availability

Individual participant data will not be available in accordance with the General Data Protection Regulation. A de-identified dataset including the data analyzed during the current study may however be available from the corresponding author on reasonable request.

## Abbreviations

ANCOVA: Analysis of covariance
BFP: Body fat percentage
BMI: Body mass index
CI: Confidence interval
CVD: Cardiovascular disease
DXA: Dual-energy x-ray absorptiometry
ECG: Electrocardiogram ES Effect size
FBG: Fasting blood glucose
FM: Fat mass
HAI: Healthy Ageing Initiative
HDL: High-density lipoprotein cholesterol
IQR: Interquartile range
LBM: Lean body mass
LDL: Low-density lipoprotein cholesterol
RPE: Rate of perceived exertion
SE: Supervised exercise
TC: Total cholesterol
TG: Triglycerides
VAT: Visceral adipose tissue
WE: Web-based exercise
