# Supplementary material for: Web-based exercise versus supervised exercise for decreasing visceral adipose tissue in older adults with central obesity: a randomized controlled trial
Source: BMC Geriatr. 2020 May 12;20:173. doi: 10.1186/s12877-020-01577-w (PMC7216357; doi:10.1186/s12877-020-01577-w)
Supplement: Supplementary file 3 — Additional file 3. Written description of the exercises used in the training program. [file 12877_2020_1577_MOESM3_ESM.docx]

**Description of exercises**


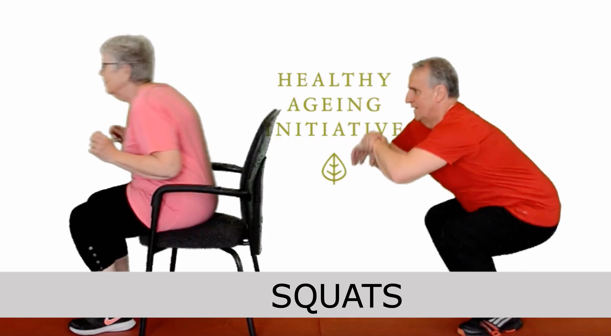


**Squats**

**Base**: Sit on a chair with both feet placed firmly in the ground. From this position, initiate the movement by standing up as quickly and explosively as possible. Once you’ve reached full hip- and knee extension sit back down once again. Find a suitable tempo that you’re able to sustain throughout the duration of the interval. If you need support, feel free to use the armrests. If this is the case, a goal for you during future sessions may be to perform the movement with less or no support from the armrests. If you don’t use the armrests you can activate your upper body by tying your hands together in front of you as you sit down and extend them along the side of your body as you stand up.

**Variation**: For progression, you can increase the tempo, minimize pauses at the extended position, and use less of the support from the armrests. If you want to challenge yourself, you can try performing a free squat without use of the chair.


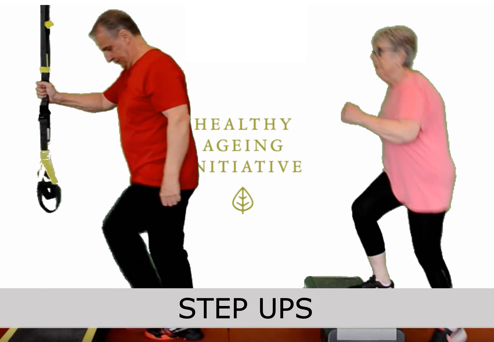


**Step ups**

**Base**: Stand below a staircase, ramp or similar. Make sure you have something close to use for support such as the staircase railing or the training straps. From here, you’re going to climb up and down on the ramp in a as high of a tempo as you feel you can manage.

**Variation**: For variation and progression you may as you take a step up on the ramp life that knee upwards, flexing the hip. Another alternative is to simply increase the tempo, let go of the hand support, and/or swing your arms as you step up and down.


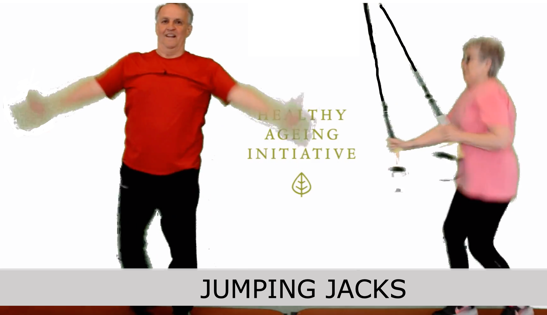


**Jumping jacks**

**Base**: Stand with your feet and arms together. Start by taking one step aside with your right foot as far as you can, while at the same time moving your arms up over your head. Continue by returning to the middle, bringing down your arms, and then moving on to your other side repeating the same motion and from here alternate between left and right throughout the duration of the interval.

**Variation**: To challenge yourself, either increase the tempo performing the above-described motion or jump with both feet outward simultaneously for an even more challenging movement. If you experience shoulder pain, do not reach your arms fully above your ears, or bring them out in front of you instead. If you experience knee pain, jump with the feet in the sagittal plane to reduce the stress on the knees. If necessary, use the suspension bands for support. For progression, increase the tempo.


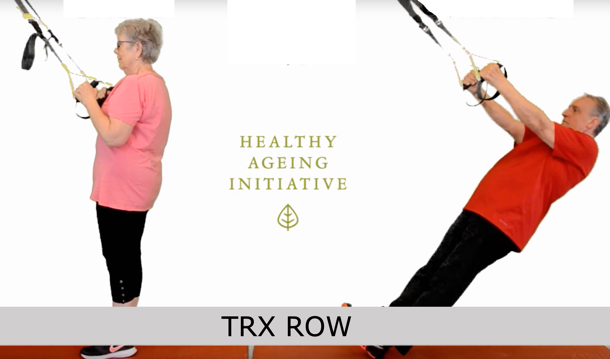


**TRX row**

**Base**: Start by grabbing hold of the straps and extending your arms in front of you with your thumbs pointing up. Initiate the movement by pulling the straps towards your chest and actively retracting your shoulder blades. Once your elbows are in line with, or slightly behind your body, pause for a split second before extending your arms back to the starting position in a controlled manner.

**Variation**: To challenge yourself, progressively increase the inclination towards the ground.


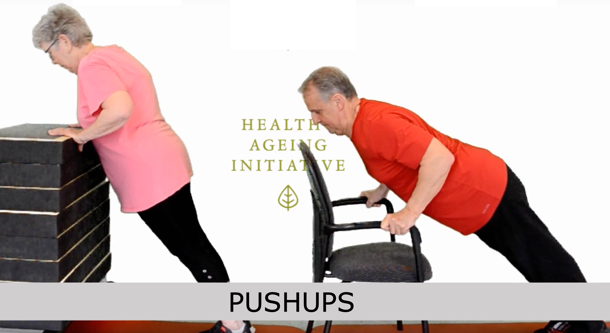


**Pushups**

**Base**: Stand easily leaning forward at a window sill, table or something similar at the appropriate height. Place your hands just outside shoulder width, or slightly wider. Initiate the movement by flexing at the elbow joint and in a breaking motion get your chest as close as possible to the window sill. Once there, push back to the extended starting position, and repeat this motion pattern for the duration of the interval

**Variation**: For progression, increase the inclination by placing the feet further backward or by finding a lower object/platform to push against

**
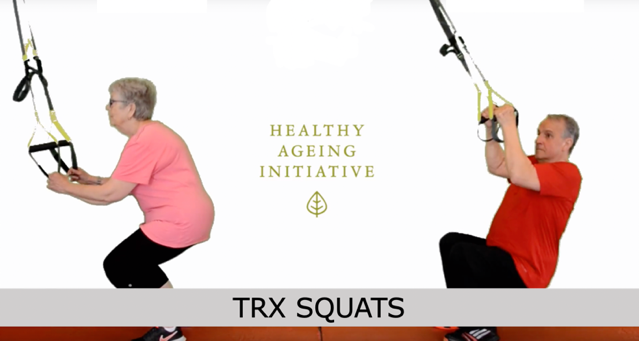
**

**TRX squats**

**Base**: Start by standing with a slight backward inclination, your feet about hip width apart, and grab hold of the straps. From here, flex your hips and knees and sit down into a deep squat. Once you’ve reached an appropriate and comfortable depth, stand up again. As you accumulate fatigue, feel free to use your arms to pull yourself up.

**Variation**: Increase the tempo and minimize pauses. For an even more challenging variation you can finish each repetition by perform a vertical jump.


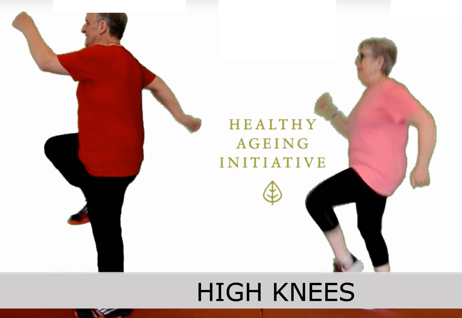


**High knees** **Base**: Lift your right knee and left arm simultaneously, and then alternate. Keep as high of a tempo as possible.  **Variation**: Increase the tempo and swing your arms in a motion similar to as if you were running.

**Sprint**

**Base**: Walk at a fast pace back and forth across the room.

**Variation**: Increase the tempo, jog or jog with high knees.
